# Disrupting the balance: how acne duration impacts skin microbiota assembly processes

**DOI:** 10.1128/spectrum.02603-24

**Published:** 2025-02-24

**Authors:** Lang Sun, Qingqun Wang, Jing Huang, Huan Wang, Zheng Yu

**Affiliations:** 1 Department of Microbiology, Human Microbiome and Health Group, School of Basic Medical Science, Central South University 12570 , Changsha, Hunan, China; 2 Department of Parasitology, School of Basic Medical Science, Central South University 12570 , Changsha, Hunan, China; 3 Department of Dermatology, The Fourth Hospital of Changsha 625085 , Changsha, China; University of Arkansas for Medical Sciences, Little Rock, Arkansas, USA

**Keywords:** microbial assembly, microbial community, acne, disease duration, microbial ecology

## Abstract

Growing interest in the impact of microbial balance on health has driven studies on the ecological processes shaping the skin microbiota. Skin diseases, which alter the skin’s local environment, can disrupt the microbial structure and interact with the disease itself. However, research on microbial assembly in diseased skin remains limited. In this study, we applied ecological models to characterize the processes shaping the skin microbiota in acne patients, considering the impact of disease duration on both skin pores and surfaces using bacterial amplicon sequencing. Our results revealed a significant shift in microbial diversity on the skin surface of patients with long-term acne. Further microbial community analyses showed a transition in ecological processes from healthy to diseased skin. Microbial communities on the skin surfaces of healthy controls and individuals with short-duration acne were primarily driven by heterogeneous selection, whereas microbial drift dominated the assembly process in the long-duration groups. Using the Sloan neutral model, we classified amplicon sequence variants (ASVs) into high-effect and low-effect groups based on relative abundance and sample occurrence. High-effect ASVs, likely exerting a greater ecological influence, were predominantly represented by *Cutibacterium* across all acne-affected skin groups, while *Staphylococcus* became enriched among high-effect ASVs in patients with long-term acne. Functional profiling further demonstrated that high-effect ASVs were significantly enriched in motility-related pathways. Additionally, we observed a reduction in microbial network complexity on skin surfaces as disease duration increased. Overall, the ecological dynamics of skin microbial communities may offer valuable insights into the mechanisms underlying disease onset and persistence.

**IMPORTANCE:**

The skin microbiota plays a critical role in acne development, yet the processes governing microbial assembly during acne progression remain poorly understood. Previous studies predominantly focused on factors such as acne severity, location, and duration in relation to skin microbial structure, with little attention given to the ecological mechanisms shaping the communities. In this study, we applied ecological models to investigate the processes influencing microbial assembly of skin microbiota in acne patients with varying disease durations and examined functions of ecologically important non-neutral amplicon sequence variants (ASVs). Our findings reveal a transition in ecological processes from deterministic to neutral processes as acne duration increased, with non-neutral ASVs potentially contributing to acne pathogenicity and persistence. These insights contribute to a deeper understanding of the ecological dynamics underlying acne and indicate that targeting these non-neutral ASVs or their associated functions may serve as the basis for future therapeutic strategies.

## INTRODUCTION

Skin is the largest organ of the human body that harbors various microbial organisms, including bacteria, fungi, and viruses. Skin-associated microbial communities play an important role in protecting the host against pathogens, regulating the immune system, and aiding in the digestion of organic compounds (1). Healthy adults can maintain stable skin microbiota for at least 2 years, despite ongoing environmental changes (2). Conversely, dysbiosis in this delicate microbial balance is frequently associated with skin diseases, such as eczema (3), acne (4), and chronic wounds (5). There is increasing interest in selecting or designing microbial communities to influence host health or shape host functions (6). This underscores the importance of understanding the forces that shape and maintain these host-associated microbial communities.

Acne is a chronic inflammatory skin disease that arises from skin pores, affecting approximately 85% of adolescents and 11% of adults (7). It often persists or recurs within individuals from adolescence to adulthood, with manifestations ranging from asymptomatic to disfigurement (8, 9). Mental health and quality of life in acne patients can be significantly influenced (10). Research on the human skin microbiome during acne has revealed that specific phylotypes or strains of *Cutibacterium acnes* are commonly associated with the disease (11, 12). In addition, in severe or prolonged cases, *Staphylococcus* can outcompete *Cutibacterium*, indicating dynamic shifts in microbial composition during the disease (13, 14). Shifts in skin microbiota during acne may be driven by host or environmental factors. This represents a valuable model for disentangling skin microbial assembly processes during the diseased state, with potential implications for health interventions.

Ecological models have been widely used to discern the forces driving microbial community assembly (15). The neutral theory posits that community structure is determined by stochastic forces, including birth, death, colonization, extinction, and speciation, without the influence of species characteristics, such as passive dispersal and ecological drift (16). In contrast, the niche-based theory hypothesizes that deterministic forces, such as species characteristics, microbial interactions, environmental filtering, and host immunity, could influence microbial community structure and fitness (17, 18). Recent studies have applied these theories to better understand and predict normal skin microbial communities. For example, skin microbial assembly varied by city population size, with megacities dominated by niche processes and smaller cities by stochastic processes (19). Following a rapid environmental change (i.e., piercing), the skin microbiome was predominantly stochastic but became more deterministic over time (20). Temporal dynamics of the skin mycobiome were dominated by stochastic processes (21). However, the ecological processes influencing skin microbial assembly in disease states such as acne, are not yet well understood.

Here, we collected a total of 70 skin samples from acne lesions, consisting of 31 skin pores and 39 skin surface samples, from 18 acne patients and 4 health controls, which were then subjected to 16S rRNA gene amplicon sequencing. This study aimed to apply ecological models to analyze the processes shaping the skin microbiota in acne patients with varying disease durations. Specifically, we sought to assess the relative contributions of niche-based and neutral processes in influencing the microbial community composition in patients with short and long disease durations; construct co-association networks to explore potential microbial interactions; and examine differences in predicted functions between amplicon sequence variants (ASVs) that are selectively favored or disadvantaged within the skin microbiota during acne.

## RESULTS

### Diversity of skin microbial communities

Seventy skin samples were collected from 22 subjects: 31 from skin surfaces (out) and 39 from skin pores (in). All samples were rarefied to the sample with the lowest number of reads (54,642 reads), and the rarefaction curves (Fig. S1) demonstrate that the sequencing depth was sufficient to capture the majority of microbial diversity, as all curves plateaued. To evaluate the grouping criteria for downstream microbial assembly process analysis, beta diversity comparisons were performed with individual patients blocked to minimize single-patient variability. Sampling location combining disease duration exhibited significant differences based on both Bray-Curtis and weighted UniFrac distances as shown in principal coordinate analysis (PCoA) plots (Fig. S2). In the downstream analyses, samples were grouped into the following five groups: health_out (skin surfaces from health controls), long_in (skin pores from long-duration acne), short_in (skin pores from short-duration acne), long_out (skin surfaces from long-duration acne), and short_out (skin surfaces from short-duration acne).

**Fig 1:**
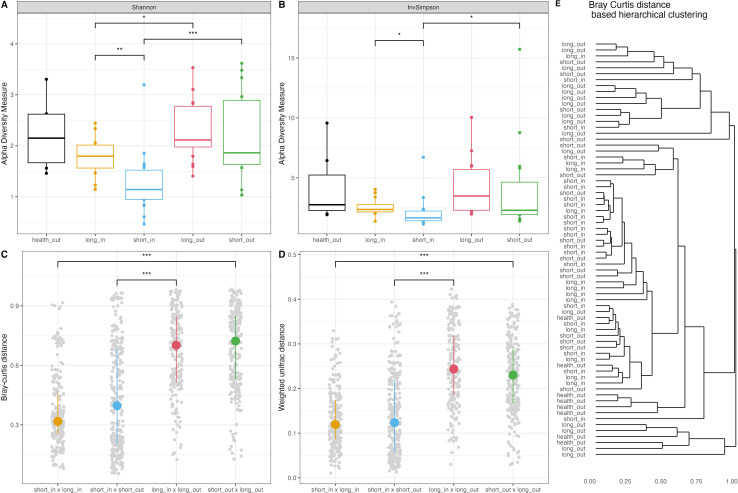
Microbial community diversities in skin pore and surface samples across short and long acne durations. Shannon (**A**) and InvSimpson (**B**) indices of microbial communities. Pairwise comparisons between groups based on Bray-Curtis (**C**) distance and weighted Unifrac (**D**) distance. (**E**) Hierarchical clustering based on Bray-Curtis distance for all skin samples. Sample groups: health_out (skin surfaces from health controls), long_in (skin pores from long-duration acne), short_in (skin pores from short-duration acne), long_out (skin surfaces from long-duration acne), and short_out (skin surfaces from short-duration acne). Levels of significance were calculated using the Wilcoxon test and are indicated by asterisks: * *P* < 0.05, ** *P* < 0.01, and *** *P* < 0.001.

Alpha diversity was primarily higher in skin surface samples compared to skin pore samples with the same acne duration (Fig. 1A and B). Additionally, the alpha diversity of skin pores was significantly higher in the long-duration group compared to the short-duration group, as shown by both Shannon and Inverse Simpson indices. However, no significant differences in alpha diversity were observed between healthy controls and acne groups, or among acne durations on skin surfaces. Beta diversity dissimilarities, based on Bray-Curtis and weighted Unifrac distances, were pairwise compared between sample groups (Fig. 1C and D). The greatest dissimilarities in communities were observed on skin surfaces between short and long acne durations (short_out vs long_out), followed by “long_in vs long_out,” “short_in vs short_out,” and “short_in vs long_in.” Hierarchical clustering based on Bray-Curtis distance revealed that long_out samples were largely separated from all other samples, while there were similarities and overlaps in the microbial compositions among the remaining sample groups (Fig. 1E). These results suggest that the divergence in bacterial community composition was most pronounced on the skin surface with long-term acne duration, though the distinction between some of the other sample groups was less pronounced.

### Microbial community assembly with ecological models

**Fig 2:**
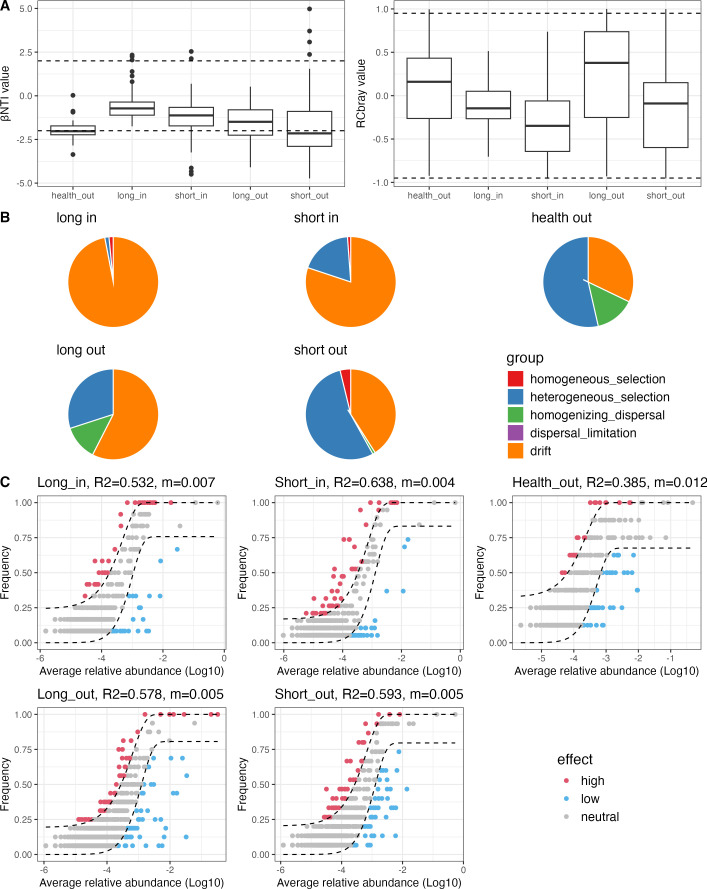
Microbial community assembly analyses. (**A**) βNTI and Raup-Crick index (RCbray) values based on the null model. (**B**) Relative proportions of varying ecological processes based on the null model. (**C**) Fit to the Sloan neutral model. Dashed lines represent 95% confidence intervals of the model prediction. ASVs above, below, or within the prediction are displayed in red, blue, and gray, respectively. Sample groups: health_out (skin surfaces from health controls), long_in (skin pores from long-duration acne), short_in (skin pores from short-duration acne), long_out (skin surfaces from long-duration acne), and short_out (skin surfaces from short-duration acne).

To understand the variations in skin microbial structure, ecological models were applied to analyze the processes governing microbial community assembly in acne skin samples. The β Nearest Taxon Index (βNTI), calculated based on a null model, was used to assess the balance between deterministic and stochastic processes. The mean βNTI values in the long_in (−0.56), short_in (−1.23), and long_out (−1.61) groups all fell within the neutral range, suggesting that these bacterial communities were predominantly shaped by stochastic processes. Additionally, RCbray values in these groups were within the (−0.95, 0.95) range, indicating that drift played a significant role in this stochastic process. In contrast, the health_out and short_out groups exhibited a potential deterministic process with a mean βNTI value of −2.02 and −2.01, respectively, suggesting that skin surface samples from patients with a short duration of acne might be influenced by environmental selection (Fig. 2A).

As shown in Fig. 2B, pie charts were used to depict subprocesses of the microbial community assembly. In skin pores, drift was the predominant subprocess, increasing with disease duration (80% in short_in, 97% in long_in). Heterogeneous selection also contributed, with a higher impact in the short_in group (19%) compared to the long_in group (1.5%). Homogeneous selection remained low and consistent across both groups (1% in short_in, 1.5% in long_in). On skin surfaces, drift and heterogeneous selection were the main subprocesses. As acne duration increased, the percentage of drift also increased (32% in health_out, 41% in short_out, and 58% in long_out), while the percentage of heterogeneous selection decreased (54% in health_out, 54% in short_out, and 30% in long_out). Homogenizing dispersal was more pronounced in both health_out (14%) and long_out group (13%) compared to the short_out group (1%), while homogeneous selection was only notable in the short_out group (4%). These results underscore the significant role of stochastic processes in shaping skin microbiota during acne, with selective pressures having a greater impact on skin surfaces with short-duration acne or in healthy skin.

**Fig 3:**
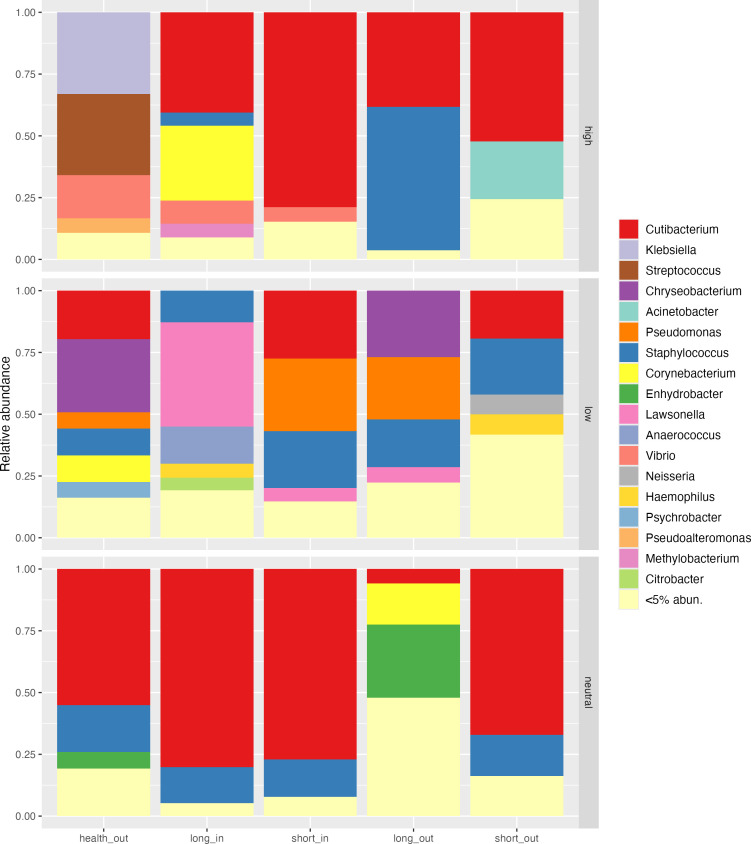
Relative abundance of bacterial genera across different Sloan model effect partitions. Columns represent sample groups: health_out (skin surfaces from health controls), long_in (skin pores from long-duration acne), short_in (skin pores from short-duration acne), long_out (skin surfaces from long-duration acne), and short_out (skin surfaces from short-duration acne). Rows indicate the Sloan model effect categories: high-effect, low-effect, and neutral.

Next, we used the Sloan neutral model to investigate whether ASVs in the skin microbial community follow stochastic or deterministic processes. An R² value greater than 0.5 indicates that stochastic processes predominate, while ASVs deviating from the prediction interval were categorized as either high-effect or low-effect, depending on whether they appeared more or less frequently than expected by the model. High-effect ASVs are likely to have a more significant ecological role within the community, often associated with growth advantages or dominance in the environment, while low-effect ASVs typically play a more marginal role, exhibiting less prominence in terms of growth or influence in the community. As shown in Fig. 2C, the neutral model fit well for microbial communities across all acne-associated groups, whereas health_out exhibited a deterministic effect. We then examined non-neutral ASVs in different skin samples to understand the taxa responses (Fig. 3). *Cutibacterium* exhibited a relatively high abundance across all the high-effect partition of acne-associated groups, underscoring its general ecological advantage and significance in the acne-associated skin environment. In contrast, *Staphylococcus* showed increased abundance in the high-effect partition of the long_out group, while *Corynebacterium* was prominent in the high-effect partition of the long_in group. These findings suggest that prolonged acne duration may create distinct growth advantages for *Staphylococcus* and *Corynebacterium*, shaped by their respective ecological niches. Interestingly, *Vibrio*, though not typically associated with the skin microbiome, was detected in the high-effect partitions of health_out, as well as short_in and long_in groups. Meanwhile, *Lawsonella*, *Chryseobacterium*, and *Pseudomonas* were predominantly identified in low-effect partitions, likely due to their high abundance in only a few samples, limiting their overall impact on the broader communities (Fig. S3).

### Predicted functions of skin microbial communities

**Fig 4:**
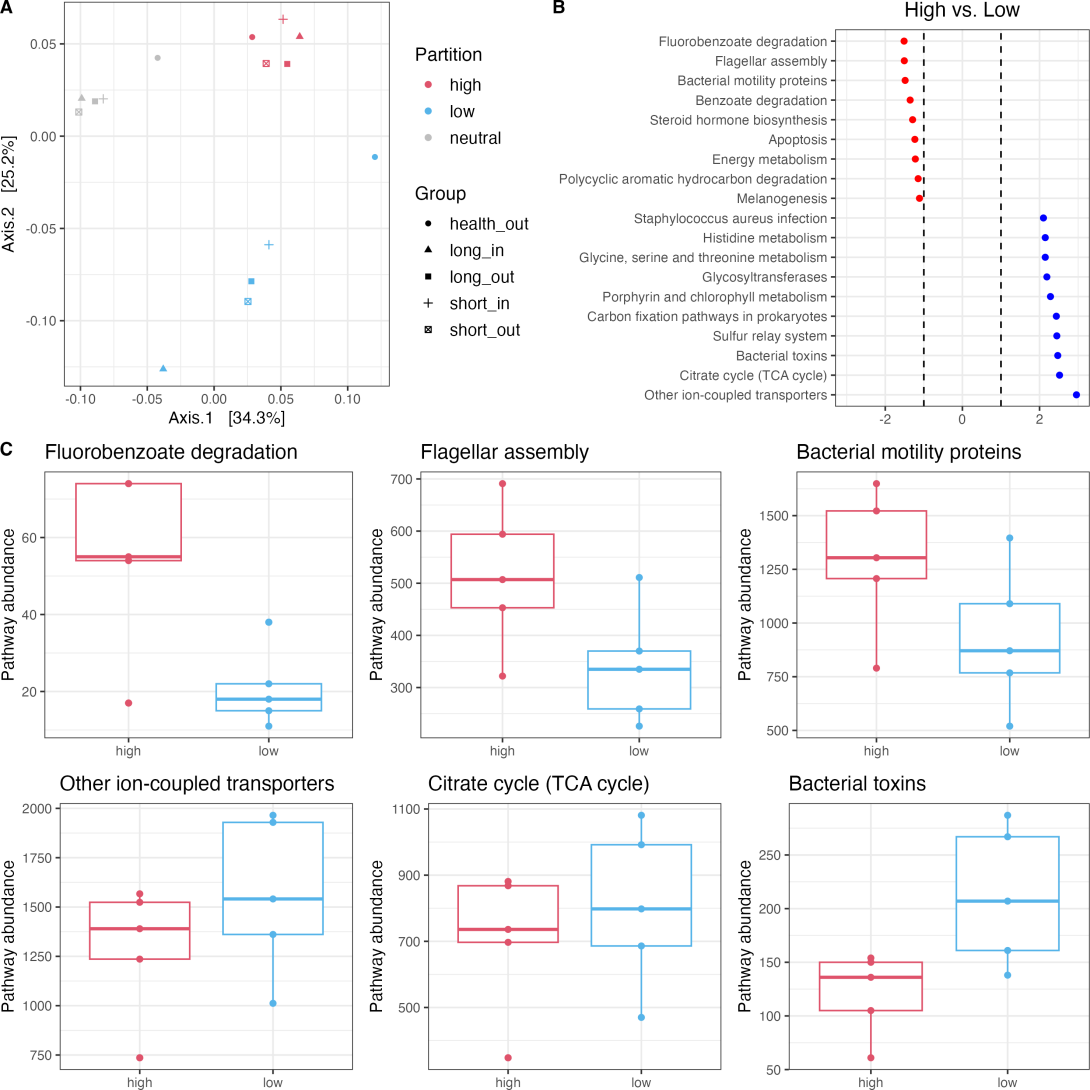
Predicted functions of ASVs above, below, or within the Sloan model prediction. (**A**) PCoA of predicted functions based on Bray-Curtis distances. Sample groups: health_out (skin surfaces from health controls), long_in (skin pores from long-duration acne), short_in (skin pores from short-duration acne), long_out (skin surfaces from long-duration acne), and short_out (skin surfaces from short-duration acne). (**B**) Differential abundance analysis of predicted functional pathways between high and low partitions identified by ALDEx2. (**C**) Boxplots of the abundance distribution of the top three differential pathways enriched in high and low partitions.

To determine whether the high-, low-, or neutral-effect partitions, as predicted by the Sloan neutral model, could be explained by their functional profiles, we conducted a Phylogenetic Investigation of Communities by Reconstruction of Unobserved States (PICRUSt2) analysis. The predicted KEGG orthology (KO) for ASVs in these partitions formed distinct clusters based on Bray-Curtis distance, regardless of disease duration or sample location (Fig. 4A). This suggests that ASVs within each partition share similar functional profiles, which may offer insights into the ecological partitions observed in the model.

ALEDx2 analysis identified 71 significantly different functional pathways between the high- and low-effect partitions, with the top enriched pathways in each partition displayed in Fig. 4B. ASVs in the high-effect partition were enriched in pathways related to xenobiotic degradation (e.g., fluorobenzoate, benzoate degradation, and polycyclic aromatic hydrocarbon), suggesting that the microbial communities may be adapting the skin environment by utilizing a diverse range of organic compounds. Additionally, the pathways related to flagellar assembly and bacterial motility proteins indicate that these microbes are highly mobile, which may allow them to adapt quickly to changes in the host environment or move to advantageous niches on the skin surface. In contrast, ASVs in the low-effect partition were enriched in pathways associated with metabolism and energy production (e.g., TCA cycle, carbon fixation, and amino acid metabolism) and transport and cellular functions (e.g., ion-coupled transporters, sulfur-related system, and glycosyltransferases), suggesting that these functions may not provide a competitive advantage in the context of the skin-related environment. Interestingly, functions related to bacterial pathogenesis, including bacterial toxins and *Staphylococcus aureus* infection, were also enriched in the low-effect ASVs. This suggests that while *Staphylococcus aureus* and other pathogenic bacteria are present, the skin generally maintains mechanisms to limit their ecological influence. However, as selective forces diminish with increasing acne duration, the reduced capacity to restrict potential pathogens may allow them to thrive on the skin over time and may contribute to disease progression. The exact abundances of represented pathways in both partitions are shown in Fig. 4C.

### Networks of skin microbial communities

**Fig 5:**
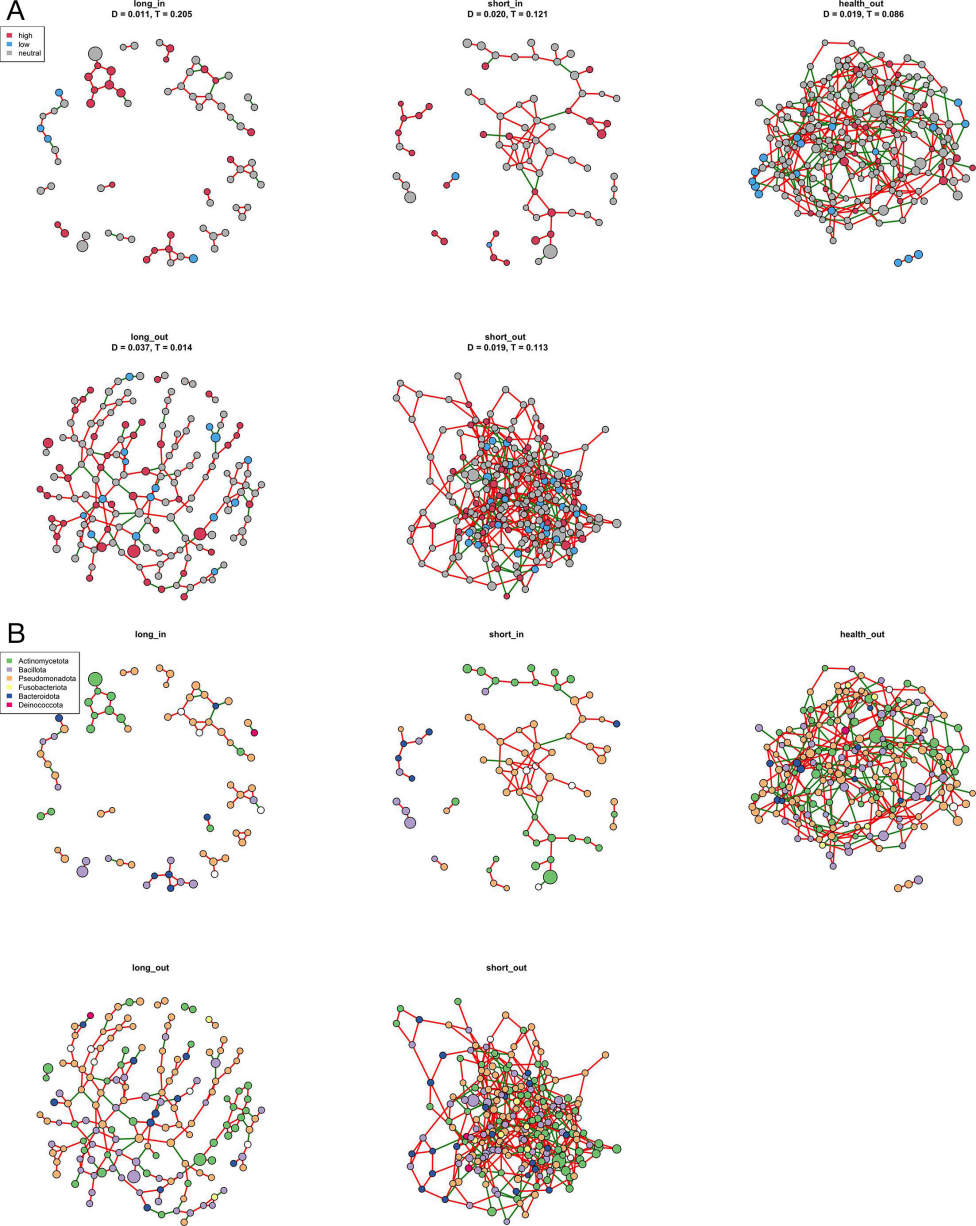
Co-association network in skin samples. (**A**) Nodes are color-coded according to neutral model partitions. (**B**) Nodes are color-coded based on bacterial phylum. The size of each node is proportional to the relative abundance of each ASV. The red and green edges represent positive and negative correlations, respectively. Sample groups: health_out (skin surfaces from health controls), long_in (skin pores from long-duration acne), short_in (skin pores from short-duration acne), long_out (skin surfaces from long-duration acne), and short_out (skin surfaces from short-duration acne).

To understand the interaction relationships and structures of skin microbiota, co-association networks were constructed (Fig. 5). In the network, density (D) measures the level of interconnectedness within the microbial community, while transitivity (T) reflects the probability that interconnected microbes form cohesive clusters. Overall, skin surfaces exhibited higher density but lower transitivity compared to skin pores, indicating that while bacterial interactions on skin surfaces are more numerous, they are less cohesive or stable. Specifically, the long_in group had a slightly higher density compared to the short_in group, suggesting a greater number of interactions among bacteria in skin pores during long-term acne. Conversely, the long_out group showed lower density and transitivity compared to the health_out and short_out groups, implying a less stable and more fragmented network of bacterial interactions on the skin surface in long-term acne. These findings suggest that the microbial network structure varies with both the duration of acne and the location on the skin, which could influence the development and persistence of acne.

Nodes in the network were color-coded based on Sloan neutral model partitions (Fig. 5A) and bacterial phylum (Fig. 5B). Interactions were more frequent between bacteria from the neutral and high-effect partitions, potentially explaining their dominance in skin microbial communities compared to those in the low-effect partition. Additionally, positive correlations were predominantly found within the same phylum, particularly among *Actinomycetota*. Taxa from *Actinomycetota*, such as *Cutibacterium* and *Corynebacterium*, exhibited strong mutual positive connections, indicating that *Actinomycetota* species may play a crucial role in maintaining microbial networks on the skin.

## DISCUSSION

Community assembly involves the gathering, colonization, and interaction of native and immigrant species to form a local community (22). Various factors influence shifts in microbial community structure, including local niches, host immunity, interactions between microbiota and hosts, and genetic differences (23–26). While some studies have explored microbial community assembly on healthy human skin, understanding this process in diseased skin is equally important, as it can provide insights into the role of microbiota in disease progression and treatment. In the present study, we applied ecological models to analyze the microbiome assembly processes in skin samples from acne patients, focusing on short- and long-duration groups. Our findings revealed significant differences in microbial structures between these groups, particularly on the skin surfaces. The ecological models indicated that skin microbial communities during acne predominantly follow neutral processes. However, stronger selective forces were observed on the skin surfaces of healthy controls and the short-duration acne group. Furthermore, the predicted functions of non-neutral ASVs provided insights into the selective forces acting on the skin microbial communities.

The variation in microbial diversity was most notable in the long_out group, likely influenced by the lower microbial diversity in skin pores and the cumulative impact of changes in microbial structure on skin surfaces. This gradual change in microbial communities is consistent with previous observations during puberty acne, in which beta diversity significantly differs between disease and healthy controls over time (12). It’s possible that as acne progresses, certain microbial communities are better adapted to the altered skin environment.

Our results indicate that the assembly of skin microbiota showed a transition in ecological processes from healthy to diseased skin. Microbial assembly in long-duration acne is primarily driven by neutral processes, suggesting that random events like microbial dispersal, colonization, and local extinction play a major role. Deterministic processes, which involve selective forces acting on specific bacterial communities, appear to be more prominent in the skin surfaces of healthy individuals and the short-duration acne group. Unlike the gut, the skin offers limited nutrients, making it a less hospitable environment for microbial survival and growth. In addition to scarce nutrients, the skin’s dry conditions, fluctuating temperature, exposure to UV radiation, and host immune responses further limit microbial persistence (1). These factors collectively impose selective pressures on the microbial community. As previous research suggests, acne can alter the skin’s microenvironment, including pH (27), moisture (28), and sebum composition (29), which in turn may influence the selective forces that either favor or eliminate certain taxa. This microenvironment change may involve a shift from commensal to more pathogenic microbial species. Beyond these abiotic factors, biotic interactions may also play a crucial role in shaping microbial communities. Skin commensals have long been recognized for their vital role in maintaining the skin barrier and participating in host immune regulation (30–32). However, it remains unclear whether the diseased state of the skin, such as in acne, disrupts these microbial interactions and the balance they maintain. In the present study, our results also revealed that the ecological dynamics in the skin’s surface and deeper layers are distinct. Drift emerged as the dominant process in skin pores, while dispersal was only observed on skin surfaces. These differences may be associated with the anatomical structure of skin pores, which experience fewer environmental disturbances (33).

The Sloan neutral model analysis indicates that *Staphylococcus* is typically found in the neutral- and low-effect partition of most skin microbial communities. Additionally, functional pathways related to *Staphylococcus aureus* infection were also enriched in the low-effect partition. These findings suggest a competitive or regulatory process that prevents certain *Staphylococcus* ASVs from dominating the community, likely due to host immune defenses or interactions with more dominant and protective commensals (34). However, the shift of *Staphylococcus* to dominance in the high-effect partition with prolonged acne duration suggests a reduction in these limiting processes, which aligns with the decreasing influence of deterministic processes, as indicated by the assembly model. These *Staphylococcus* ASVs identified in the high-effect partition during long-duration acne may play an important role in acne progression and persistence. Our study highlights that certain *Staphylococcus* may initially act as invaders during the early stages of acne development with difficulties in colonizing among skin commensals. However, they may eventually demonstrate resilience by successfully thriving within the skin communities, particularly in cases of prolonged acne. *Staphylococcus* is ubiquitous on the skin and comprises different species, including *S. epidermidis*, *S. hominis*, *S. haemolyticus*, *S. capitis*, *S. aureus*, and others (1). Notably, different *Staphylococcus* spp. or even within a species show functional differences that could help maintain skin microbiota balance or facilitate skin infections (35). It points to the need for more comprehensive strain-level investigations within *Staphylococcus*, as these functional differences may separate commensal or pathogenic *Staphylococcus*. More efforts are needed to characterize the strain-specific functional profiles of *Staphylococcus* and their role in modulating the host immune response and inflammation, which are still challenging with amplicon sequencing technology (36).

Furthermore, we observed that the stability of microbial networks decreased with prolonged acne duration on skin surfaces. This decreased stability may help explain why acne often relapses or persists in patients with long-term acne, as previous studies have shown that lower network stability makes microbial communities more vulnerable to environmental disturbances (1). Additionally, this decreased stability may indicate a shift in microbial interactions from beneficial symbiosis to dysbiosis. Future research aimed at restoring a more stable microbiome or enhancing microbial network resilience may benefit skin health, as has been proposed for gut health. Strategies such as microbiota transplantation, supplementation with probiotics and prebiotics, or modification with microbial nutrients have shown promise in restoring balance to gut microbial communities (37). Additionally, consistent with the previous observations in the skin mycobiome (21), microbial interactions tend to occur among microbes from the same phylum. This phenomenon may be related to particular environmental conditions that favor the retention of microbes sharing similar traits essential for environmental adaptation, as reported in many other natural microbial communities (38, 39). These findings also open the door for potential targeted interventions aimed at rebalancing the skin microbiota by promoting beneficial microbes while inhibiting acne-associated species.

In summary, our study revealed the bacterial community assembly process both in the skin pores and on the surfaces across varying acne durations. The deterministic process was more pronounced on skin surfaces of healthy controls and the short-duration acne group, as determined by the null model and Sloan neutral model. However, the skin microbiota of acne patients with long-duration acne was predominantly driven by the stochastic process. Differential functional pathways between ASVs from high- and low-effect partitions were notably enriched in bacterial motility and infection pathways, respectively. It is important to note that functional predictions based on 16S rRNA gene sequencing may not fully capture the true functional diversity of the microbiome. To gain a more comprehensive understanding of microbial community functions, future research should incorporate metagenomics and metatranscriptomics. One limitation of this study is the small sample size in the long-duration acne group, along with multiple samples taken from the same subject, which may influence the robustness of the findings. Future studies with larger sample sizes are needed to validate these results further. Overall, our findings offer valuable insights into the microbial assembly process in skin disorders, enhancing our understanding and prediction of skin microbial communities.

## MATERIALS AND METHODS

### Subject recruitment and sample collection

Diagnosis of acne, subject recruitment, and sample collection were conducted as previously described (13). In brief, subjects with acne were recruited at the Fourth Hospital of Changsha, Changsha, Hunan, China. Patients with acne were diagnosed by dermatologists based on the Pillsbury grading scale (40). Subjects with the following circumstances were excluded from the study: oral or topical antibiotics, retinoids, glucocorticoids, immunosuppressants, and other agents within the past month; any other skin or chronic diseases; lactating or pregnant women. Acne duration was determined by patients filling out the questionnaire at the hospital or referring to previous clinical history records. The entire process was guided by dermatologists. A total of 70 skin samples were collected with 31 samples from skin pores and 39 from surfaces from facial areas (Table S1). Skin surface samples were collected using sterile cotton swabs (Bo di fu, Lanxi, China) soaked with 0.9% NaCl and 0.1% Tween 20 solution (TS) at a 2.5 × 2.5 cm^2^ area. The area for skin pore sampling was first sterilized with 75% medical alcohol (Hynaut, Qingdao, China), and contents in the skin pores were collected by puncturing and pressing hair follicles with acne needles. The negative control was also collected during each visit to the hospital to ensure that no contamination occurred during the sampling process. All samples were transferred to the laboratory within 4 h on ice. After adding 1 mL TS, swabs were vortexed for 8 min and supernatants were centrifuged for 5 min at 12,000 rpm. Then, supernatants were discarded, and pellets were kept at −80°C until DNA extraction. The informed consent and questionnaire were signed by all participants voluntarily. This study (IRB No. 2021-KT75) was approved by the Ethical Review Board of the School of Basic Medical Science at Central South University and conducted in adherence to the Declaration of Helsinki Principles.

### DNA extraction and amplicon sequencing

Cetyltrimethylammonium bromide (CTAB) was used to extract genomic DNA from skin samples (13). In brief, CTAB (Sangon Biotech, Shanghai, China), lysozyme (Sangon Biotech, Shanghai, China), protease K (Sangon Biotech, Shanghai, China), and 20% SDS were added for cell lysis. Phenol (Macklin, Shanghai, China), chloroform (Macklin, Shanghai, China), and isoamyl alcohol (Macklin, Shanghai, China) were then added for protein precipitation. Samples were centrifuged at 12,000 rpm for 10 min to separate the aqueous phase (containing DNA) from the organic phase and precipitated proteins. Isopropanol (Macklin, Shanghai, China), glycogen (Sangon Biotech, Shanghai, China), and sodium acetate (Sangon Biotech, Shanghai, China) were then added and incubated at −20°C for 30 min for DNA precipitation. Precipitated DNA was collected after centrifugation at 12,000 rpm for 10 min and then cleaned using 75% ethanol (Sangon Biotech, Shanghai, China) and air-dried. After being rehydrated in DNase-free water, the quality of the DNA was assessed using the NanoDrop ND-1000 spectrophotometer (Thermo Fisher Scientific, Waltham, USA). Primer pair (515F: 5′-GTGCCAGCMGCCGCGGTAA-3′/806R: 5′-GGACTACHVGGGTWTCTAAT-3′) were used to amplify the 16S rRNA V4 region. This region was chosen for the study because it provides a longer overlap during sequencing, which helps to reduce the error rate. Sample-specific 7 bp barcodes were incorporated into the primers for multiplex sequencing. The PCR components contained 5 µL of buffer (5×), 0.25 µL of Fast pfu DNA Polymerase (5 U/µL), 2 µL (2.5 mM) of dNTPs, 1 µL (10 uM) of each Forward and Reverse primer, 1 µL of DNA template, and 14.75 µL of ddH_2_O. Thermal cycling conditions included 98°C for 2 min and 25 cycles (98°C for 15 s, 55°C for 30 s, and 72°C for 30 s), and a final extension of 5 min at 72°C. Amplified PCR products were purified using Vazyme VAHTSTM DNA Clean Beads (Vazyme, Nanjing, China). All purified PCR products were further quantified with the Quant-iT PicoGreen dsDNA Assay Kit (Invitrogen, Carlsbad, USA), and sequenced on the Illumina MiSeq platform. Pair-end (2 × 250 base pair) sequencing was conducted according to the NovaSeq 6000 SP Reagent Kit (500 cycles) (Illumina, San Diego, USA). DNA in negative controls was also extracted, and no detectable amplification was observed in the negative controls, confirming that contamination was not introduced during sampling or processing.

### Sequence preprocessing and data analysis

The resulting raw sequences were processed with the DADA2 package (v. 1.28.0) in R (v. 4.3.0) (41, 42). Bacterial forward and reverse sequences were trimmed at 220 bp and 160 bp based on sequencing quality figures. Filtered sequences were then inferenced, merged, and chimera removed according to the DADA2 pipeline. Bacteria taxonomic assignment was based on the Silva database (v. 138.2) (43). Species-level assignment was further analyzed by BLASTn ASV sequences against the standard database (nt/nr). ASVs aligning with archaea, eukaryotes, chloroplasts, and mitochondria were removed. Alpha diversity was measured by Shannon and Inverse Simpson indices using the phyloseq package (v. 1.44.0) (44). Beta diversity was measured as Bray-Curtis and weighted Unifrac distances.

βNTI based on phylogenetic diversity and Raup-Crick based on Bray-Curtis taxonomic diversity (RC_bray_) were used to estimate the relative contribution of neutral and niche-based processes (22). Briefly, abundance-weighted β mean nearest-taxon distance (βMNTD) was calculated to quantify the phylogenetic turnover of microbial community structure using the picante package (v. 1.8.2) (45). Then, a predicted βMNTD was calculated based on a null model with 999 randomizations, and deviations between predicted and observed βMNTD were represented by βNTI. The proportion less than the observed Bray-Curtis distance was represented by the RC_bray_ by comparing the predicted and observed distance. The absolute value of βNTI > 2 or βNTI < −2 represents homogeneous selection and heterogeneous selection, respectively. |βNTI| < 2 with |RCbray| < 0.95 suggests the drift contribution, and |βNTI| < 2 with RC_bray_ > 0.95 or RC_bray_ < −0.95 indicates dispersal limitation and homogenizing dispersal, respectively. Relative contributions of subprocesses were further quantified by the null model (46).

Skin microbial communities were then fit to the Sloan neutral model using the MicEco package (v. 0.9.15) (47). The model compared predicted occupancy–abundance values for each ASV with the observed ASV values in sample groups. ASVs that occurred within the 95% confidence interval are neutral taxa, while ASVs that occurred more frequently (high-effect) or less frequently (low-effect) than prediction are considered as actively selected or excluded taxa. Functions with high-effect and low-effect partitions were further predicted using PICRUSt2 (48). In brief, ASVs were positioned within a phylogenetic tree alongside full reference genomes of 16S rRNA, corrected by 16S rRNA copy number per genome, and function-predicted based on the Kyoto Encyclopedia of Genes and Genomes (KEGG) database (49). Distribution of predicted functions in three partitions was visualized using PCoA. Then, KEGG ortholog pathways at level 3 were extracted for further differential abundance analysis. The ALDEx2 package (v. 1.30.0) was used to perform differential abundance analysis of functions between high- and low-frequency partitions (50). A difference in effect size ≥1 with a significant *P* value < 0.05 based on Wilcoxon’s rank test or Welch’s *t*-test was identified.

Co-association networks were constructed based on the neighborhood algorithm with a minimum λ threshold of 0.01 using the SPIEC-EASI (v. 1.1.2) (51) software and visualized using the igraph package (v. 1.4.2) (52). The density (D) of a network represents the ratio between the number of edges and the number of all potential connections in a network. The degree of clustering between a node’s neighboring nodes is defined as transitivity (T).

## Data Availability

Except for data related to subject characteristics, raw sequence data in this study have been deposited in the NCBI SRA under accession no. PRJNA995545.

## SUPPLEMENTAL MATERIAL

Table S1 and Figures S1 to S3 spectrum.02603-24-s0001.docx Table S1: Skin sample information. Figure S1: Rarefaction curves. Figure S2. Principal coordinate analysis (PCoA) of samples. Figure S3. Microbial composition of skin microbiota.

ASM does not own the copyrights to Supplemental Material that may be linked to, or accessed through, an article. The authors have granted ASM a non-exclusive, world-wide license to publish the Supplemental Material files. Please contact the corresponding author directly for reuse.
