# Supplementary material for: Disrupting the balance: how acne duration impacts skin microbiota assembly processes
Source: Microbiol Spectr. 2025 Feb 24;13(4):e02603-24. doi: 10.1128/spectrum.02603-24 (PMC11960176; doi:10.1128/spectrum.02603-24)
Supplement: Table S1 and Figures S1 to S3 — Table S1: Skin sample information. Figure S1: Rarefaction curves. Figure S2. Principal coordinate analysis (PCoA) of samples. Figure S3. Microbial composition of skin microbiota. [file spectrum.02603-24-s0001.docx]

**Supplementary Material**

**Table S1 Skin sample information.**

| **Subject ID** | **Gender** | **Sample ID** | **Age** | **Acne duration** | **Duration Group** | **Location** |
| --- | --- | --- | --- | --- | --- | --- |
| S1 | male | CA1 | 16-20 | 1-3y | long | out |
| S1 | male | CA2 | 16-20 | 1-3y | long | in |
| S2 | female | CA10 | 21-25 | <1y | short | out |
| S2 | female | CA11 | 21-25 | <1y | short | in |
| S3 | female | CA100 | 21-25 | <1y | short | in |
| S3 | female | CA95 | 21-25 | <1y | short | out |
| S4 | female | CA103 | 21-25 | <1y | short | out |
| S4 | female | CA104 | 21-25 | <1y | short | out |
| S4 | female | CA105 | 21-25 | <1y | short | out |
| S4 | female | CA107 | 21-25 | <1y | short | in |
| S4 | female | CA108 | 21-25 | <1y | short | in |
| S5 | male | CA13 | 16-20 | 1-3y | long | out |
| S5 | male | CA14 | 16-20 | 1-3y | long | in |
| S6 | female | CA15 | 16-20 | 4-6y | long | out |
| S6 | female | CA16 | 16-20 | 4-6y | long | in |
| S7 | male | CA17 | 16-20 | 1-3y | long | out |
| S7 | male | CA20 | 16-20 | 1-3y | long | out |
| S7 | male | CA21 | 16-20 | 1-3y | long | out |
| S7 | male | CA22 | 16-20 | 1-3y | long | out |
| S7 | male | CA25 | 16-20 | 1-3y | long | in |
| S7 | male | CA26 | 16-20 | 1-3y | long | in |
| S7 | male | CA27 | 16-20 | 1-3y | long | in |
| S8 | female | CA29 | 21-25 | <1y | short | out |
| S8 | female | CA31 | 21-25 | <1y | short | in |
| S8 | female | CA32 | 21-25 | <1y | short | in |
| S9 | male | CA3 | 16-20 | 1-3y | long | out |
| S9 | male | CA6 | 16-20 | 1-3y | long | out |
| S9 | male | CA7 | 16-20 | 1-3y | long | in |
| S10 | female | CA34 | 21-25 | <1y | short | out |
| S10 | female | CA36 | 21-25 | <1y | short | out |
| S10 | female | CA37 | 21-25 | <1y | short | in |
| S10 | female | CA38 | 21-25 | <1y | short | in |
| S11 | male | CA42 | 16-20 | <1y | short | in |
| S11 | male | CA43 | 16-20 | <1y | short | in |
| S12 | female | CA44 | 16-20 | 1-3y | long | out |
| S12 | female | CA45 | 16-20 | 1-3y | long | out |
| S12 | female | CA46 | 16-20 | 1-3y | long | out |
| S12 | female | CA47 | 16-20 | 1-3y | long | out |
| S12 | female | CA48 | 16-20 | 1-3y | long | in |
| S12 | female | CA51 | 16-20 | 1-3y | long | in |
| S13 | male | CA54 | 16-20 | <1y | short | out |
| S13 | male | CA57 | 16-20 | <1y | short | in |
| S13 | male | CA58 | 16-20 | <1y | short | in |
| S13 | male | CA59 | 16-20 | <1y | short | in |
| S14 | female | CA65 | 16-20 | <1y | short | in |
| S15 | female | CA68 | 21-25 | <1y | short | out |
| S15 | female | CA69 | 21-25 | <1y | short | in |
| S15 | female | CA70 | 21-25 | <1y | short | in |
| S16 | female | CA71 | 21-25 | 4-6y | long | out |
| S16 | female | CA72 | 21-25 | 4-6y | long | out |
| S16 | female | CA73 | 21-25 | 4-6y | long | out |
| S16 | female | CA74 | 21-25 | 4-6y | long | in |
| S16 | female | CA75 | 21-25 | 4-6y | long | in |
| S16 | female | CA76 | 21-25 | 4-6y | long | in |
| S17 | male | CA77 | 16-20 | <1y | short | out |
| S17 | male | CA80 | 16-20 | <1y | short | out |
| S17 | male | CA81 | 16-20 | <1y | short | out |
| S17 | male | CA83 | 16-20 | <1y | short | out |
| S17 | male | CA84 | 16-20 | <1y | short | in |
| S17 | male | CA89 | 16-20 | <1y | short | in |
| S18 | female | CA90 | 21-25 | <1y | short | out |
| S18 | female | CA93 | 21-25 | <1y | short | in |
| S19 | female | N102 | 21-25 | 0y | health | out |
| S20 | female | N118 | 21-25 | 0y | health | out |
| S20 | female | N119 | 21-25 | 0y | health | out |
| S20 | female | N120 | 21-25 | 0y | health | out |
| S20 | female | N121 | 21-25 | 0y | health | out |
| S21 | female | N123 | 26-30 | 0y | health | out |
| S21 | female | N124 | 26-30 | 0y | health | out |
| S22 | female | N30 | 21-25 | 0y | health | out |

**
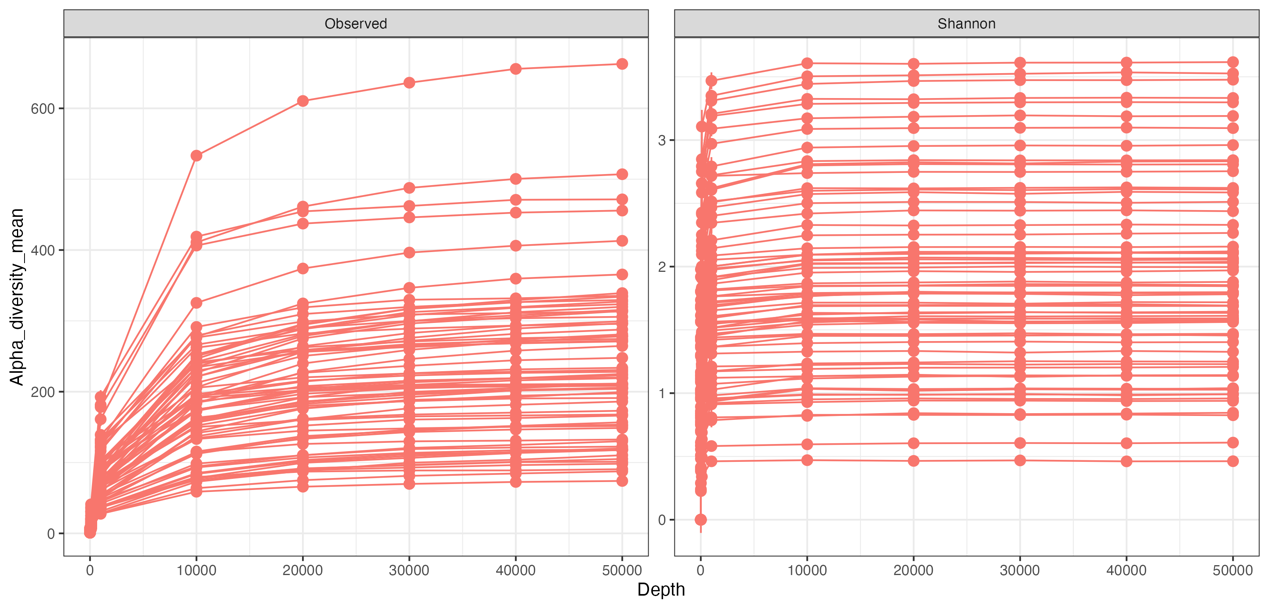
**

Figure S1. Rarefaction curves showing the relationship between sequencing depth and observed microbial diversity across all samples.


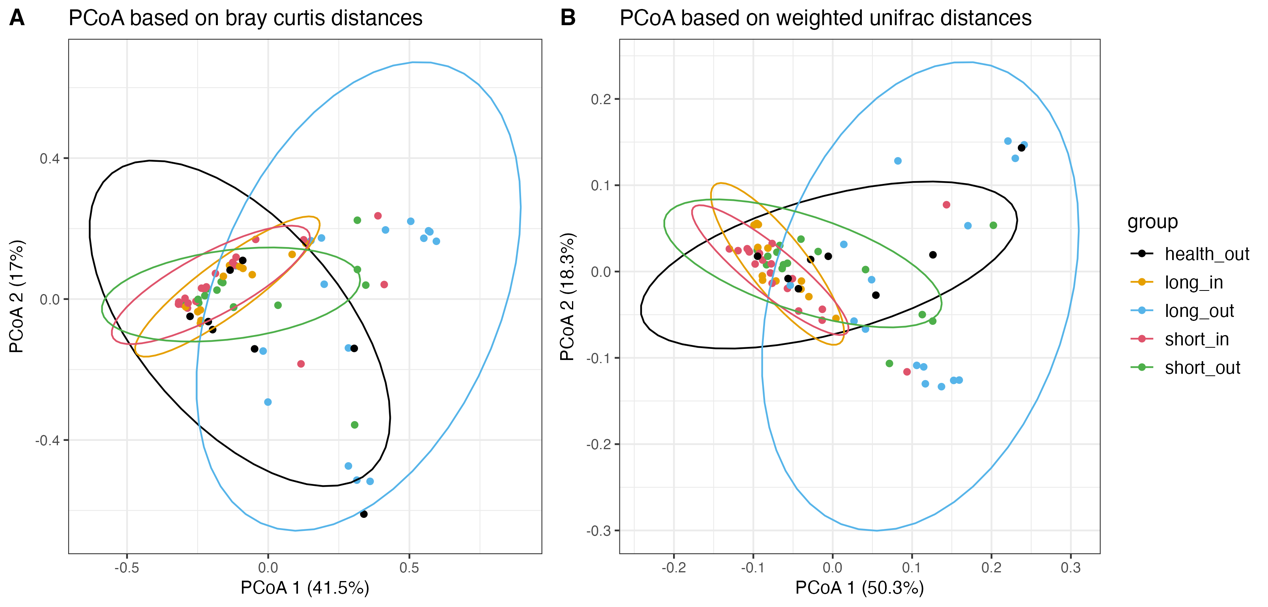


Figure S2. Principal coordinate analysis (PCoA) of samples grouped by location and disease duration. PERMANOVA analysis, blocked by individual subject, revealed distinct clustering based on the current grouping (*p* < 0.001). Sample groups: health_out (skin surfaces from health controls), long_in (skin pores from long-duration acne), short_in (skin pores from short-duration acne), long_out (skin surfaces from long-duration acne), and short_out (skin surfaces from short-duration acne).


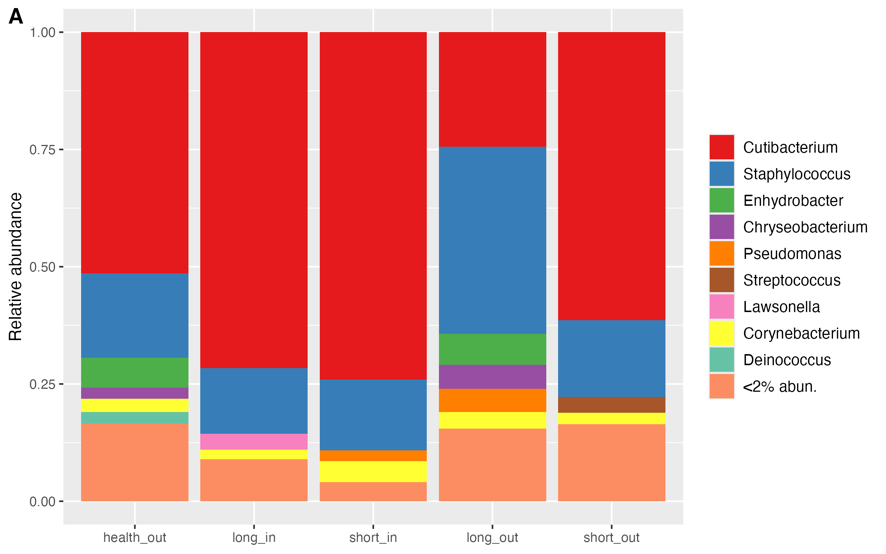

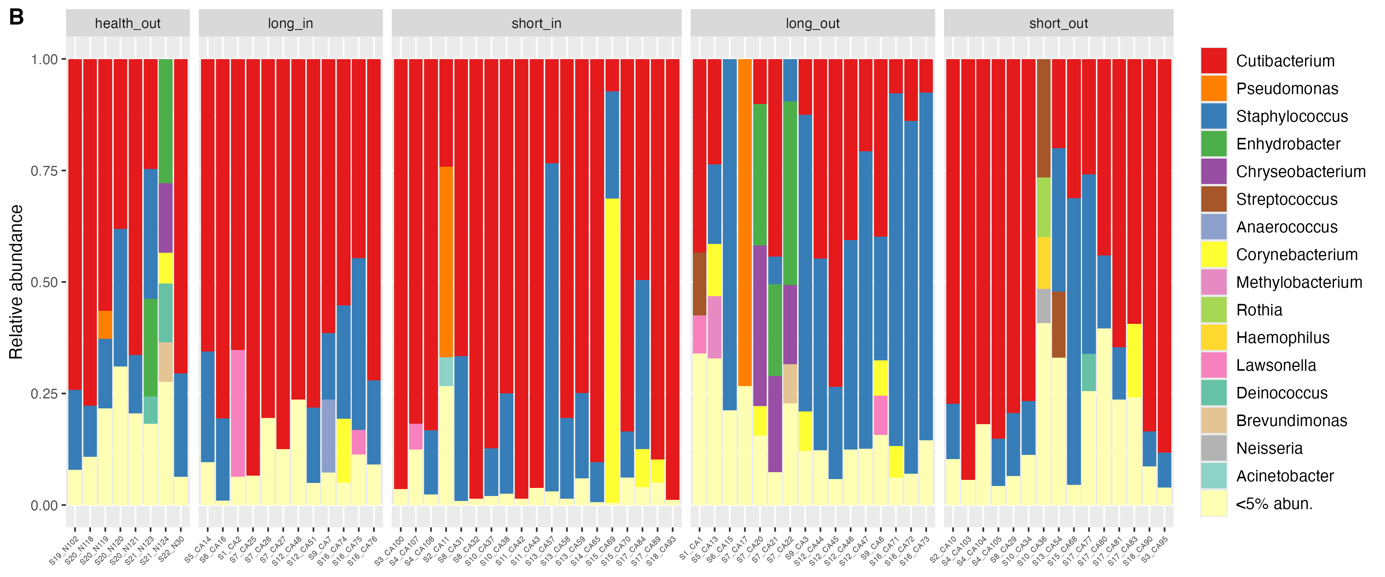


Figure S3. Microbial composition of skin microbiota for sample groups (A) and individual sample (B, x-axis was labeled by subject ID and sample ID). Sample groups: health_out (skin surfaces from health controls), long_in (skin pores from long-duration acne), short_in (skin pores from short-duration acne), long_out (skin surfaces from long-duration acne), and short_out (skin surfaces from short-duration acne).
